# Phylogenomics of the gray-breasted sabrewing (*Campylopterus largipennis*) species complex in the Amazonia and Cerrado biomes

**DOI:** 10.1590/1678-4685-GMB-2023-0331

**Published:** 2024-08-05

**Authors:** Jean Carlo Pedroso de Oliveira, Gustavo Sebastián Cabanne, Fabrício Rodrigues Santos

**Affiliations:** 1 Universidade Federal de Minas Gerais, Instituto de Ciências Biológicas, Departamento de Genética, Ecologia e Evolução, Belo Horizonte, MG, Brazil.; 2 División de Ornitología, Museo Argentino de Ciencias Naturales “Bernardino Rivadavia” (MACN - CONICET), Buenos Aires, Argentina.

**Keywords:** Hummingbirds, Neotropics, phylogeography, landscape genomics, Campylopterus

## Abstract

The Neotropics are one of the most biodiverse regions of the world, where environmental dynamics, climate and geology resulted in a complex diversity of fauna and flora. In such complex and heterogeneous environments, widely distributed species require deep investigation about their biogeographic history. The gray-breasted sabrewing hummingbird *Campylopterus largipennis* is a species complex that occurs in forest and open ecosystems of South America, including also high-altitude grasslands. It has been recently split into four distinct species distributed in Amazonia (rainforest) and Cerrado (savanna) biomes with boundaries marked by ecological barriers. Here, we investigated the evolutionary dynamics of population lineages within this neotropical taxon to elucidate its biogeographical history and current lineage diversity. We used a reduced-representation sequencing approach to perform fine-scale population genomic analyses of samples distributed throughout Amazonia and Cerrado localities, representing all four recently recognized species. We found a deep genetic structure separating species from both biomes, and a more recent divergence between species within each biome and from distinct habitats. The population dynamics through time was shown to be concordant with known vicariant events, isolation by distance, and altitudinal breaks, where the Amazon River and the Espinhaço Mountain Range worked as important barriers associated to speciation.

## Introduction

Neotropical ecosystems have attracted the worldwide attention of scientists since the dawn of evolutionary biology in the XIX century. The Neotropics hold a large portion of Earth’s biodiversity, extending from southern Mexico to as far south as central Argentina and Chile. Besides, four out of the ten most biodiverse countries worldwide are located in South America due to its particular biogeographic history. The South American landscapes have been constantly reshaped, particularly since the Eocene, but underwent significant transformation around 12 million years ago (Ma) due to the rise of the Andes and formation of the current Amazon River. These changes have contributed to the evolution of complex ecosystems within remarkably distinct biomes such as the Amazonia and the Cerrado (Hoorn et al., 2010), both of which shelter a rich and endemic biodiversity.

The Cerrado is a highly heterogeneous Neotropical savanna, and considered a global biodiversity hotspot (Myers et al., 2000; Colli et al., 2020). Besides typical savannas, it also presents grassland fields, woody forests, gallery forests, and patches of Seasonally Dry Tropical Forests (SDTFs) (Prado and Gibbs, 1993; Oliveira-Filho et al., 2006; Neves et al., 2015). The heterogeneous ecosystems of the Cerrado biome are also connected with the Amazonia biome in central Brazil, fostering important interactions (Marques et al., 2020).

The Amazonia biome includes also many heterogeneous landscapes that are mostly composed of forest ecosystems. The historical environment dynamics in Amazonia have led to high levels of regional diversification and endemism, making it the primary source of Neotropical biodiversity (Antonelli et al., 2018), being considered a relic of a once more extensive environment (Musher et al., 2019). The species richness in the Amazon region has also been associated with high rates of *in situ* speciation and lineage sharing (Hoorn et al., 2010; Smith et al., 2014) due to facilitated emigration (Musher *et al*., 2019) and the presence of areas of endemism (Ribas et al., 2012; Braga et al., 2022).

Climate change is known to be an important cause of vegetational changes over large areas (Haffer, 1969; Cheng et al., 2013). The Amazonia and Cerrado biomes have experienced intense biogeographic interaction, mostly related to Quaternary Climatic Fluctuations (Werneck, 2011). Also, the Pleistocene Arc Hypothesis (PAH) postulates that dry forests enclaves had a broader and more contiguous distribution during the Pleistocene glacial maxima, but became fragmented and reduced during interglacial periods, as observed in the present (Prado and Gibbs, 1993; Pennington et al., 2000; Neves et al., 2015). These dynamics of expansion and retraction of dry forests during the Pleistocene might have resulted in a heterogeneous landscape of open and forest-savanna biomes between Amazonia and Cerrado (Marques et al., 2020). Consequently, this interaction engendered distinct biogeographic patterns and phylogenetic relationships among species (Werneck *et al*., 2012; Rocha et al., 2020), including also the sharing of bird taxa between the two biomes (Vasconcelos and D’Angelo, 2018).

Birds drive diverse hypotheses on biota diversification in the Neotropics (Ribas et al., 2012; Musher et al., 2019; Silva et al., 2019; Norambuena and Van Els, 2021). For example, connections between Neotropical forests have been deeply explored and different forest corridors through the dry diagonal biomes (Cerrado, Caatinga and Chaco) promoted gene flow in multiple bird taxa (Batalha-Filho et al., 2013; Cabanne et al., 2016; Trujillo-Arias et al., 2017; Capurucho et al., 2018; Berv et al., 2021). Thus, bird dispersal enables gene flow between distant populations from different biomes and ecosystems (Lima-Rezende et al., 2019; de Freitas et al., 2022). Indeed, the Cerrado biome has been indicated as a provider of corridors for bird dispersal through riparian forests connecting Amazon and Atlantic forests (Batalha-Filho *et al*., 2013; Ledo and Colli, 2017; Trujillo-Arias *et al*., 2017 ; Cabanne *et al*., 2019), where some related species of rainforest taxa have established within Cerrado. Ledo and Colli (2017) revisited the connections between tropical forests, stating that the central Brazilian riparian network was an important route between Amazonia and Cerrado, but not as much with Atlantic Forest. This strengthens the hypothesis of the ability of bird species to succeed in occupying intermediate or new habitats, such as along the headwaters of Paranã River (Tocantins basin), and Jequitinhonha and Doce rivers (Willis, 1992; Capurucho *et al*., 2018).

**Figure 1-:**
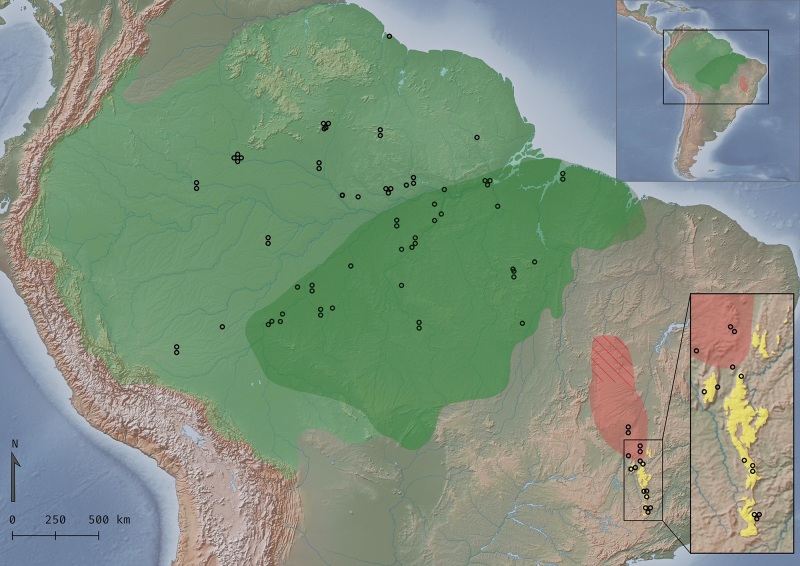
Geographic distribution of samples used in the phylogenomic analysis of the *Campylopterus largipennis* species complex. Polygons represent species ranges *Campylopterus largipennis* (green), *C. obscurus* (light green*), C. calcirupicola* (red: extant; hatched: possibly extant) and of *C. diamantinensis* (yellow). Distributions adapted from BirdLife International (BirdLife International, 2021) and Lopes et al. (2017). The inset indicates the study area in South America and markers indicate sampled individuals.

The gray-breasted sabrewing species complex represents a unique case to investigate lineage diversification in the Neotropics, and particularly between Amazonia and Cerrado biomes and ecosystems. Until recently, it was recognized a single widespread species, *Campylopterus largipennis,* that occupied diverse ecosystems of Amazonia and Cerrado. With the recent description of *Campylopterus calcirupicola* (Lopes et al., 2017), the gray-breasted sabrewing species complex taxonomy was revised after a century of uncertainty by the International Ornithological Committee (Gill *et al*., 2021) and the Brazilian Ornithological Records Committee (Pacheco et al., 2021). Two species are currently recognized in the Cerrado biome: *Campylopterus calcirupicola* Lopes, Vasconcelos & Gonzaga, 2017 that inhabits the SDTFs (*Matas Secas*), and *C. diamantinensis* Ruschi, 1963 endemic to high-altitude grassland rock outcrops (*Campo Rupestre*). Two species are recognized in the Amazonia biome: *Campylopterus largipennis* (Boddaert, 1783) that inhabits southern/southeastern Amazonia, and *C. obscurus* Gould, 1848, that inhabits northern/western Amazonia (Figure 1).

Many competing hypotheses about taxa diversification between Neotropical ecosystems have been reevaluated with genomic evidence (Baker et al., 2014), since the large amount of data generated by massive sequencing increased the robustness of biogeographic analyses. Considering this, the four closely-related hummingbird species of the *C. largipennis* complex represent a unique case to investigate ancient lineage diversification between Neotropical ecosystems and biomes, as their genetic signatures in a continent-wide distribution may reconstruct their history of successful occupation of Amazonia and Cerrado.

Here, we investigate the population dynamics and biogeographical history of the gray-breasted sabrewing *Campylopterus largipennis* species complex. The broad distribution of these four closely related taxa in distinct Amazonia and Cerrado ecosystems offers a unique opportunity to evaluate the long-term processes of population establishment and divergence in response to diverse environments. We used genome-wide SNPs identified by double digest restriction associated DNA sequencing (ddRAD) method to evaluate population genetic structure, demographic history, gene flow and isolation patterns. Our study aims to elucidate the evolutionary pathways of these populations within the Amazonia and Cerrado biomes through phylogeographic and landscape genetics analyses, thereby addressing hypotheses regarding genetic differentiation, habitat connectivity, and biogeographic implications.

## Material and Methods

### Sample collection

We assessed a total of 78 individuals distributed along Amazonia and Cerrado biomes (Figure 1, Table S1), covering almost the entire distribution of the *Campylopterus largipennis* species complex, representing all four recognized taxa. All samples were previously available in the public collections of Centro de Coleções Taxonômicas of Universidade Federal de Minas Gerais (CCT-UFMG), Coleção de Recursos Genéticos of Instituto Nacional de Pesquisas da Amazônia (INPA), Museu de Zoologia of Universidade de São Paulo (MZUSP) and Museu Paraense Emílio Goeldi (MPEG).

### ddRAD sequencing and SNP filtering

Genomic ddRAD libraries were prepared following the protocol of Thrasher et al. (2018), modified from Peterson et al. (2012). Genomic DNA was extracted with phenol:chloroform protocol (Sambrook and Russell 2006). Quality and quantifications were made on Nanodrop (Thermo Scientific™), gel agarose and Qubit (Thermo Scientific™). The digestion-ligation reactions were made with 400 ng of genomic DNA, digested with restriction enzymes *Sbf*I and *Msp*I (New England Biolabs, MA), then ligated on barcoded adapters for sample identification. We used 20 unique adapters for each library pool and size selected on PippinPrep (Sage Science, MA) for ~400 base pairs fragments. Additionally, samples were randomized between libraries, in order to mitigate technical library effects (O’Leary et al., 2018). We checked the libraries’ reliability by qPCR-based quantification using KAPA Library Quantification Kits (KK4604, Kapa Biosystems), before sending them to sequencing facilities. Finally, libraries were sequenced on Illumina Hiseq SE150 in Macrogen (South Korea) and one library on Illumina HiSeq PE150 in GenOne Biotechnologies (Brazil).

The quality of raw reads was checked by FastQC (Andrews, 2010), libraries were demultiplexed using BBMap (Available at http://sourceforge.net/projects/bbmap/) and assembled *de novo* (Figure S1) using iPyrad version 0.7.30 (Eaton and Overcast, 2020). Most of the parameters were used as default, with a cluster threshold of 0.90 selected after testing the cluster threshold ranging from 0.85 to 0.95, observing the error rates and heterozygosity (Mastretta-Yanes et al., 2015). The minimum samples per locus was set to 8, a stricter filter for adapters/primers was applied and reads were trimmed at 100 base pairs. Finally, the assemblies were evaluated by eye for overall coverage, using Matrix Condenser v.1.0 (Medeiros and Farrell, 2018) (Available at https://github.com/brunoasm/matrix_condenser/). A filter was applied to the Minor Allele Frequency (MAF) ranging from 0.05 to 0.1. This was done to assess the reproducibility of the protocol and to identify any potential confounding structures by conducting Principal Component Analysis (PCA) (Cumer et al., 2021). The main data set was filtered for MAF, indels and missingness using VCFTools version 0.1.16 (Danecek et al., 2011).

### Population structure and phylogenetics analysis

The starting point of population structure was inferred using the fineRADstructure package (Malinsky et al., 2018) with 200,000 iterations and burnin of 100,000. PCA was done on *adegenet* version 2.1.7 (Jombart and Ahmed, 2011) toolset on R version 4.2.1 (R Core Team, 2022). We also performed an individual-based clustering on STRUCTURE v.2.3.4 (Pritchard et al., 2000), with k = 2 to 6, 6 nreps, with 100,000 iterations and burnin of 50,000. Then, we have chosen the most likely k by the estimated log probability means and ΔK (Evanno et al., 2005). From then on, we considered the dataset separated into four main groups: Northern-Western Amazon (NWA), Southern-Eastern Amazon (SEA), *Campo Rupestre* (CR) and *Matas Secas* (MS). The pairwise F_ST_ was calculated on *hierfstat* version 0.5-11 (Goudet, 2005) using the function *boot.ppfst* with 1000 bootstraps to obtain confidence intervals of 95%. The identity-by-state (IBS) analysis of pairwise distances matrix from SNP data was done with SNPRelate package (Zheng et al., 2012). All packages were run on R version 4.2.1 (R Core Team, 2022). We estimated a maximum likelihood phylogenetic tree using RAxML-NG v.1.0.3 (Kozlov et al., 2019) for all samples, with a GTRgamma substitution model and 1,000 bootstrap replicates.

### Demographic history

A direct estimation of divergence times and temporal gene flow between phylogenetic groups were conducted on G-PHOCS version 1.3 (Gronau et al., 2011), on a subset of four samples per group based on cluster analysis and Matrix Condenser, with no indels and only loci shared between all samples. Three independent runs were performed with find-finetunes TRUE, standard priors and mutation rate 2.3 × 10^−9^ mutations per site per year (Smeds et al., 2016), scaled by 10^4^ (Gronau *et al*., 2011). The runs were set for 3,000,000 MCMC iterations, and approximately 30% of the initial portion of chains were discarded in Tracer version 1.7 (Rambaut et al., 2018), ensuring verification of posterior convergence and parameter values (τ and θ). 

### Estimating effective migration

We used the Estimated Effective Migration Surfaces (EEMS) method (Petkova et al., 2016) to identify barriers for gene flow over the landscape. We set the parameters for 2,000 demes, 2,000,000 MCMC iterations sampled every 9,999 iterations after a 1,000,000 burn-in. Three independent runs were conducted, the MCMC chain traces were checked, and the proposal variance values were adjusted following the manual. Finally, MCMC runs were combined for final plots using *rEEMSplots* in R, available with the *EEMS* package.

## Results

We obtained an average of 1.07 ± 0.7 million reads per sample. The main data set included 5,141 SNP variant positions (one per locus, 5% MAF, no indels and up 10% missing data), shared between 74 individuals of *Campylopterus largipennis* complex, from most of the total geographic distribution, including 25 individuals of *C. largipennis*, 34 individuals of *C. obscurus*, six individuals from *C. calcirupicola* and nine individuals from *C. diamantinensis*. Samples clustered according to geographic correspondence on PCAs using different values of MAF, without detecting library effects between the experiments. Only one sample (INPA_13625) was removed due to a large amount of missing data (see Table S1 for details).

**Figure 2 -:**
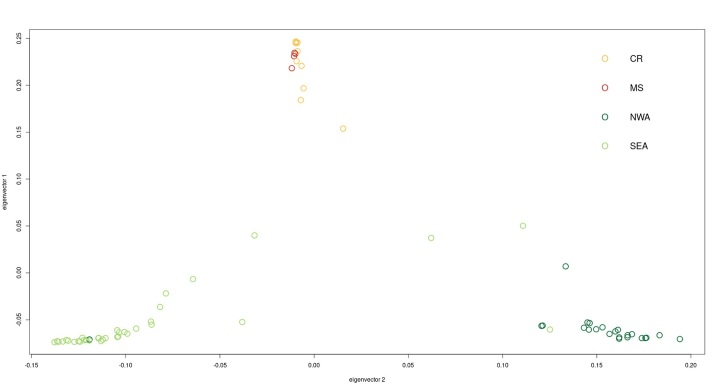
Principal components analysis (PCA) of *Campylopterus largipennis* species complex based on 3,584 SNPs. The groups are colored as follows: *C. largipennis* in green (NWA: Northern-Western Amazon), *C. obscurus* in light green (SEA: Southern-Eastern Amazon), *C. diamantinensis* in yellow (CR: *Campo Rupestre*), and *C. calcirupicola* in red (MS: *Matas Secas*).

**Figure 3-:**
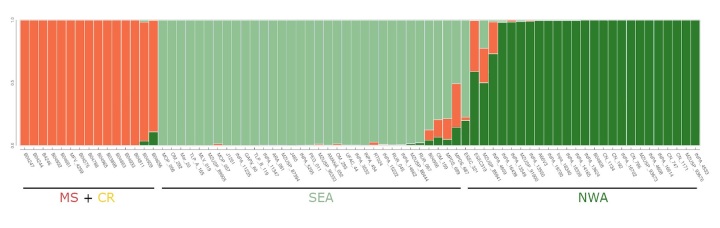
Population genetic structure of the *Campylopterus largipennis* species complex. Bayesian analysis implemented in Structure v.2.3.4, with k=3 populations estimated by ΔK and log probability means. Each bar represents an individual colored proportionally to its probability of assignment to each population. The cluster includes 74 samples of *C. calcirupicola* and *C. diamantinensis* (orange), and *C. obscurus* (green) and *C. largipennis* (light green). Location bars represent *Matas Secas* and *Campo Rupestre* (MS + CR), and Southern-Eastern (SEA) and Northern-Western (NWA) Amazon regions.

**Figure 4-:**
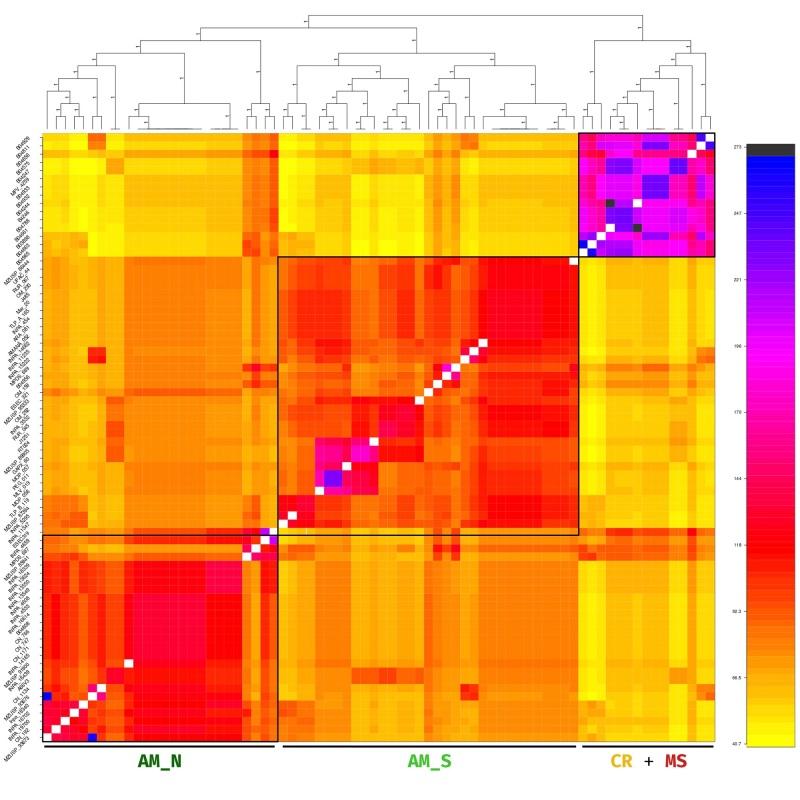
Averaged co-ancestry matrix of the *Campylopterus largipennis* species complex. Each row represents one of the 74 samples in the heatmap that indicate the degree of co-ancestry, increasing from yellow to blue.

Three major groups were identified in the data set after applying PCA, Structure, and coancestry analysis (Figures 2-4). All clustering methods showed congruence with the geographical (and biome) location of samples, revealing a closer relationship among samples from Cerrado biome (Figure 4, Figure S2). The genetic structure explained by PCA accounted for 48.5% along axis 1, separating samples from Amazonia and Cerrado, and 14.7% along axis 2, which revealed distinctions among Amazonia samples. Notably, subdivisions within the Cerrado biome only became apparent in the fourth component, indicating a relatively shallow level of differentiation (Figure S3). A hierarchical clustering analysis based on identity-by-state matrix done in *hierfstat* revealed a similar pattern observed with the clustering methods (Figure S4). The Structure analysis also detected three clusters without detecting substructure between populations from Cerrado. Most admixed individuals were located in the Southern-Western Amazon region, along the Madeira River. Although these samples present a greater admixture with the Cerrado, fineRADstructure analysis revealed a higher degree of co-ancestry between Amazonian regions. We designated these groups accordingly, naming them Northern-Western Amazon (NWA) and Southern-Eastern Amazon (SEA) groups from the Amazonia biome, separated from the Cerrado group. Following the established phylogeny (see below), we subdivided Cerrado into *Campo Rupestre* (CR) and *Matas Secas* (MS) for further analyses. Pairwise F_ST_ estimates between geographic groups ranged from 0.23 to 0.70 (Table S2) and showed significant differences between all populations.

**Figure 5 -:**
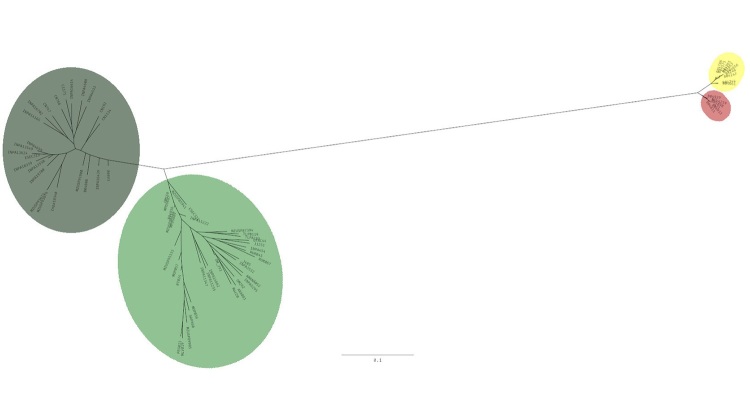
Maximum likelihood phylogenetic tree of the *Campylopterus largipennis* species complex. RAXML-NG tree generated with 4,917 loci obtained from ddRAD data filtered without indels, MAF 0.05, and missingness 90%. Colors represent *C. largipennis* in green, *C. obscurus* in light green, *C. diamantinensis* in yellow, and *C. calcirupicola* in red.

The maximum likelihood phylogenetic tree also revealed the same major groups found in clustering methods. Clades from the Amazonia and Cerrado biomes are easily distinguished (Figure 5). Further, in the Cerrado biome, CR and MS groups were both reciprocally monophyletic, even though they were not distinguished in clustering analysis (Figure 2). Besides, in Amazonia we can see South (SEA) and North (NWA) groups, even with some level of admixture found between these two clusters.

**Figure 6 -:**
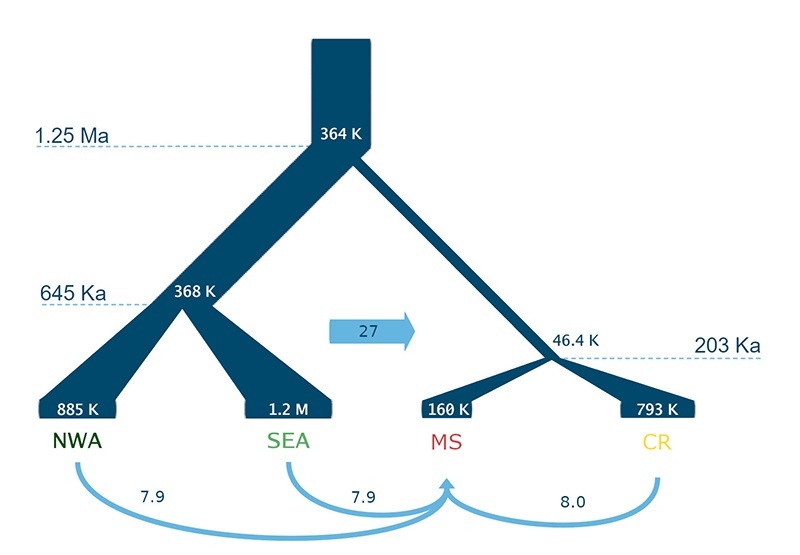
Demographic histories inferred by G-PhoCS between structured groups of *Campylopterus largipennis* (NWA) and *C. obscurus* (SEA), *C. diamantinensis* from *Campo Rupestre* (CR, montane savanna) and *C. calcirupicola* from *Matas Secas* (MS, dry-forest). Historical effective sizes are inside the tree graph, divergence times are indicated at nodes by dashed lines. Arrows indicate the number of individual migrants per generation (Msx= msx × θx/4).

**Figure 7-:**
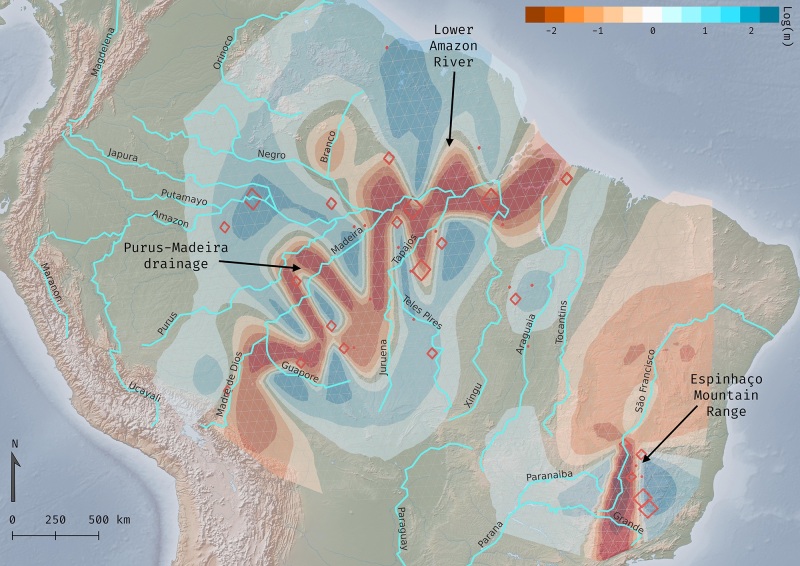
Estimated effective migration surfaces (*EEMS*) of the *Campylopterus largipennis* species complex using 2,000 demes. Red diamonds represent populations varying in size according to number of samples in the deme. Reddish areas represent barriers to migration, while bluish regions are corridors for migration. Main regional barriers to gene flow identified by *EEMS* are indicated with arrows.

G-PHOCS estimated all splitting times to different dates of milder interglacial times of the Mid-Pleistocene period (Figure 6, Table S3). The divergence between Amazonia and Cerrado lineages occurred at 1.24 Ma. The subdivisions of NWA and SEA of Amazonia occurred at 622 thousand years ago (Ka) while Cerrado clusters (CR and MS) diverged at 201 Ka. The gene flow (migration) estimates indicate an unbalanced movement of individuals (or genes) from Amazonia to Cerrado, where MS (*Matas Secas*) was the main migrant receiver. The *EEMS* analysis identified two main obstacles for gene flow (Figure 7, Table S4). The drainage system of the Purus-Madeira (upper Amazon River) is moderately permeable, but it becomes a hard barrier when it reaches the lower Amazon River, dividing current species *C. largipennis* (NWA) and *C. obscurus* (SEA). The other visible hurdle to gene flow corresponds to the altitude variance of the Espinhaço Mountain Range, which separates the highlands (CR) and lowlands (limestone SDTF environment of- MS) of Cerrado. These distinct habitats, correspond respectively to the localities of occurrence for *C. diamantinensis* and *C. calcirupicola* species.

## Discussion

Until the recent description of *Campylopterus calcirupicola*, the gray-breasted sabrewing species complex has carried taxonomic uncertainty for over a century (Lopes et al., 2017). Here we investigated a fine scale diversification of this sabrewing hummingbird species complex, which is broadly distributed across two Neotropical biomes. We assessed samples from both Cerrado and Amazonia populations, with a SNP data set covering all related taxa and most of the occurrence area. Our findings using a population genomics approach suggest a combination of landscape changes and dispersion leading to taxa diversification. While initial investigations did not identify all taxa, this may be attributed to recent speciation or potential sampling biases. Subsequent explorations have corroborated the existence of the currently recognized taxa *Campylopterus calcirupicola* Lopes, Vasconcelos & Gonzaga, 2017, *C. largipennis* (Boddaert, 1783), *C. obscurus* Gould, 1848, and *C. diamantinensis* Ruschi, 1963, reviewed by the International Ornithological Committee (Gill *et al*., 2021) and the Brazilian Ornithological Records Committee (Pacheco et al., 2021).

Gray-breasted sabrewing populations were affected by diverse biogeographic processes promoting divergence between and within biomes. The differentiation found between Amazonia and Cerrado clades were consistent with the intermediate dispersal model hypothesis (Norambuena and Van Els, 2021). Dispersal works both ways in the process of speciation. On the one hand, it can contribute to the homogenization of populations through gene flow, but it can sometimes result in the colonization of new areas, and eventually lead to subsequent local adaptation and speciation. Dispersal capacity itself is also related to geographic diversification. While species with greater dispersal capacity tend to occupy widespread areas, generating new subpopulations, species with low dispersal capacity struggle to maintain gene flow even in nearby regions. However, in both cases, the ability to disperse is related to the promotion of the speciation process. The intermediate dispersal hypothesis combines taxa dispersal ability and species diversity, and predicts that the most diverse clades are those with intermediately strong dispersal capacity (Yamaguchi, 2022). This scenario is proposed for wide distributed grassland birds in the Neotropics (Norambuena and Van Els, 2021), where dispersal is a major factor on the speciation process in a continental scale (Smith et al., 2014).

The cladogenesis events of the *Campylopterus largipennis* complex are coincident with major changes in glaciation periods. We estimated that Amazonia and Cerrado lineages separated during the Mid-Pleistocene Transition (Figure 6) at about 1.24 million years ago (Ma), the period when glacial cycles became longer and drier (Tziperman and Gildor, 2003; Willeit et al., 2019). Glacial-interglacial periods during the Quaternary can be inferred from marine oxygen-isotope stages (MIS) obtained from deep sea core samples (Wright, 2013). The subsequent division of Amazonian *C. largipennnis* and *C. obscurus* was also during a remarkably drier period approximately 621 thousand years ago (Ka), at the end of MIS 16 (Figure 6), the long-lasting cold phase of the Quaternary, characterized by very low CO_2_ atmospheric concentrations (Hughes and Gibbard, 2018). The separation of Cerrado species *C. calcirupicola* and *C. diamantinensis* occurred at the end of MIS 7, an interglacial period around 201 Ka (Figure 6). The MIS 7-6 period was relatively warmer, and the moisture supply may have allowed the formation of extensive glaciers, where the ice volume accumulated in MIS 6 is related to a global water disturbance (Hughes and Gibbard, 2018). Those colder and drier periods possibly made humid forest to shrink and open vegetation and SDTFs (Werneck, 2011) to expand in different parts of Amazonia, as registered in pollen and geochemical data (Anhuf et al., 2006; Reis et al., 2017; Wang et al., 2017; Kern et al., 2023), likely expanding suitable areas for *Campylopterus* dispersal and colonization. 

The gray-breasted sabrewings are known to be well adapted to semi-open vegetation, like margins of streams and forest edges (Schuchmann, 1999). A possible hypothesis for sabrewing hummingbird populations to have occupied the Cerrado was using forest edges in open areas as corridors for migration, since long-distance dispersal events are well known for hummingbirds. For example, the Nearctic region was invaded prior to the Panamanian uplift (<3.4 Ma) by Bee and Mountain-gem hummingbirds, followed by a rapid increase in invasions by other hummingbird lineages after the isthmus formation (McGuire et al., 2014). If somehow climate allowed the expansion to new inhabitable areas, geographical barriers worked as maintainers of local diversity. In the *EEMS* analysis (Figure 7) we can see a transition from “soft to hard” constraints for migration along the Purus-Madeira River system to the lower Amazon River, isolating *C. largipennis* (NWA) from *C. obscurus* (SEA). Most of the samples that showed some admixture degrees are from *várzea* (floodplain) areas along the Madeira River. This result also enhances the relevance of interfluves as a biogeographically important suture zone in southernmost Amazonia (Dornas et al., 2022). Similarly, samples from southeast of the Madeira and Tapajós rivers were grouped in *raxml* analysis. The resistance to migration was identified by the *EEMS* analysis between these rivers, showing to be a difficult region to cross, indicating how it has influenced the diversification of the taxon over time. Another important factor that may be contributing to the maintenance of this separation is the variability of precipitation on an orbital scale between the west and east of the Amazonia (Cheng et al., 2013). During glacial periods, this variability contributed to the greater fragmentation of forests in the eastern portion of the Amazonia.

Most of central Amazonia was supposedly occupied by SDTFs during the Pleistocene (Werneck, 2011) that were likely connected to dry forests (SDTFs) of the Cerrado, where sabrewing hummingbird ancestors could have lived and diverged into current taxa. This hypothesis is supported by a significant historical gene flow detected in G-PHOCS between Amazonia and Cerrado SDTFs (MS).

The more recent speciation of the sister species *C. calcirupicola* and *C. diamantinensis* in the Cerrado biome is likely a parapatric event related to ecological divergence in neighboring populations occupying low-altitude SDTFs/*Matas Secas* (MS) and high-altitude *Campo rupestre* (CR) on the Espinhaço Mountain Range. The occupation of different Cerrado ecosystems by two ecologically distinct sabrewing lineages likely occurred during one of the most recent glaciation-interglacial cycles of the Late Pleistocene. A possible path for the early colonization of Cerrado was through dry forests (SDTFs) and riparian forests of the headwaters of the Paranã River (Willis, 1992; Capurucho et al., 2018), which is supported by recent sabrewing records (WikiAves, 2022). 

The Neotropics were impacted by a huge and rapid event known as Great American Biotic Interchange (GABI) that ended 3.4 Ma, breaking up the continental isolation of South America since Gondwanaland split, enhancing the diversification rates (Weir et al., 2009). During the GABI, it was observed an increase in the occupation of the Nearctic region by hummingbird lineages (McGuire et al., 2014), evidencing the capacity for long dispersals by members of this group of birds. Hummingbird dispersal is also largely dependent on floral resources, acquired during a long evolutionary history. Diffuse coevolution with plants and niche conservatism is observed in hummingbird’s diversification, leading to generalists or specialist clades (McGuire *et al*., 2014). The genus *Campylopterus* belongs to Emerald hummingbirds that are usually considered generalists, being able to visit a wide variety of plants (Rodríguez-Flores et al., 2019). Indeed, the two adjacent Cerrado ecosystems (dry forest and rock outcrop fields) present completely different phytophysiognomies and are located in remarkably divergent landscapes at low (MS) and high (CR) altitudes. This generalist ecology favors distant migration and colonization of new areas, and may be related to its current distribution in a large part of Amazonia, and two particular Cerrado ecosystems.

The historical relationships between taxa from Amazonia and Cerrado are well documented for plant populations (Buzatti et al., 2018). Isolation and limited dispersal contributed to lineage diversification, and the same explain most of endemic taxa of landscapes like the high-altitude ecosystems *Campo Rupestre* and *Pantepui* (Hopper et al., 2021). Niche conservatism is observed in hummingbird mountain species that became restricted to narrow environments after colonization (McGuire et al., 2014; Rodríguez-Flores et al., 2019). Altitudinal clines also play important roles in hummingbird diversity, and genomic signatures of adaptation have been found for elevational gradients (Lim et al., 2021). Local adaptation and speciation events associated to altitude are commonly found in the Andes mountains, which hold the greatest diversity of modern hummingbirds, including other representatives of the genus *Campylopterus* (McGuire *et al*., 2014). In sabrewing populations of Cerrado, we have found an altitudinal rift between *C. diamantinensis* and *C. calcirupicola*. The *C. diamantinensis* population is restricted to high-altitude grasslands of *Campo Rupestre* and *C. calcirupicola* to the lowland SDTFs (*Matas Secas*), separated by at least 300 meters of a sudden altitudinal barrier. These species have recently diverged within the Cerrado biome, and both taxa appear to be highly adapted and limited by their specific environments (CR and MS).

Our results indicate that Pleistocenic climatic cycles and open areas dynamics likely favored the occupation by sabrewing lineages that were successful to establish in their species-specific environments. Climate fluctuations enabled the connections of such distant environments and shaped the structure of populations over time by constraining the migration between cycles. The subsequent specialization (local adaptation) to *Matas Secas* and *Campo Rupestre* allowed the maintenance of populations in these places, and lack of recent connections with the Amazon Forest favored lineage differentiation. Between MS and CR, however, the altitudinal gradient restricts gene flow between these populations. All results were congruent with the recent taxonomic revision. Here we could reconstruct successfully the historic dispersal of a generalist hummingbird species leading to the colonization of distinct environments of two major Neotropical biomes.

## Supplementary material

The following online material is available for this article:

Figure S1 - Comparative results of ddRAD runs

Figure S2 - Map with Structure results represented as individual pie charts reflecting the geographical samples distribution

Figure S3 - Paired principal component analysis. Scatterplots showing the top four PCs

Figure S4 - Hierarchical clustering analysis based on identity-by-state (IBS) matrix from SNP data representing the genetic relationships

Table S1 - Voucher specimens and location details of the samples belonging to the *Campylopterus largipennis* complex

Table S2 - Pairwise FST values sampled localities

Table S3 - Population parameters estimated from three independent runs of G-Phocs analysis

Table S4 - Migration bands from G-Phocs analysis
