# Supplementary material for: Phylogenomics of the gray-breasted sabrewing (Campylopterus largipennis) species complex in the Amazonia and Cerrado biomes
Source: Genet Mol Biol. 2024 Aug 5;47(3):e20230331. doi: 10.1590/1678-4685-GMB-2023-0331 (PMC11308382; doi:10.1590/1678-4685-GMB-2023-0331)
Supplement: Figure S1 - [file 1415-4757-GMB-47-3-e20230331-s1.pdf]

Genetics and Molecular Biology
https://doi.org/10.1590/1678-4685-GMB-2023-0331

Supplementary Material to "Phylogenomics of the gray-breasted sabrewing (*Campylopterus largipennis*) species complex in the Amazonia and Cerrado biomes"

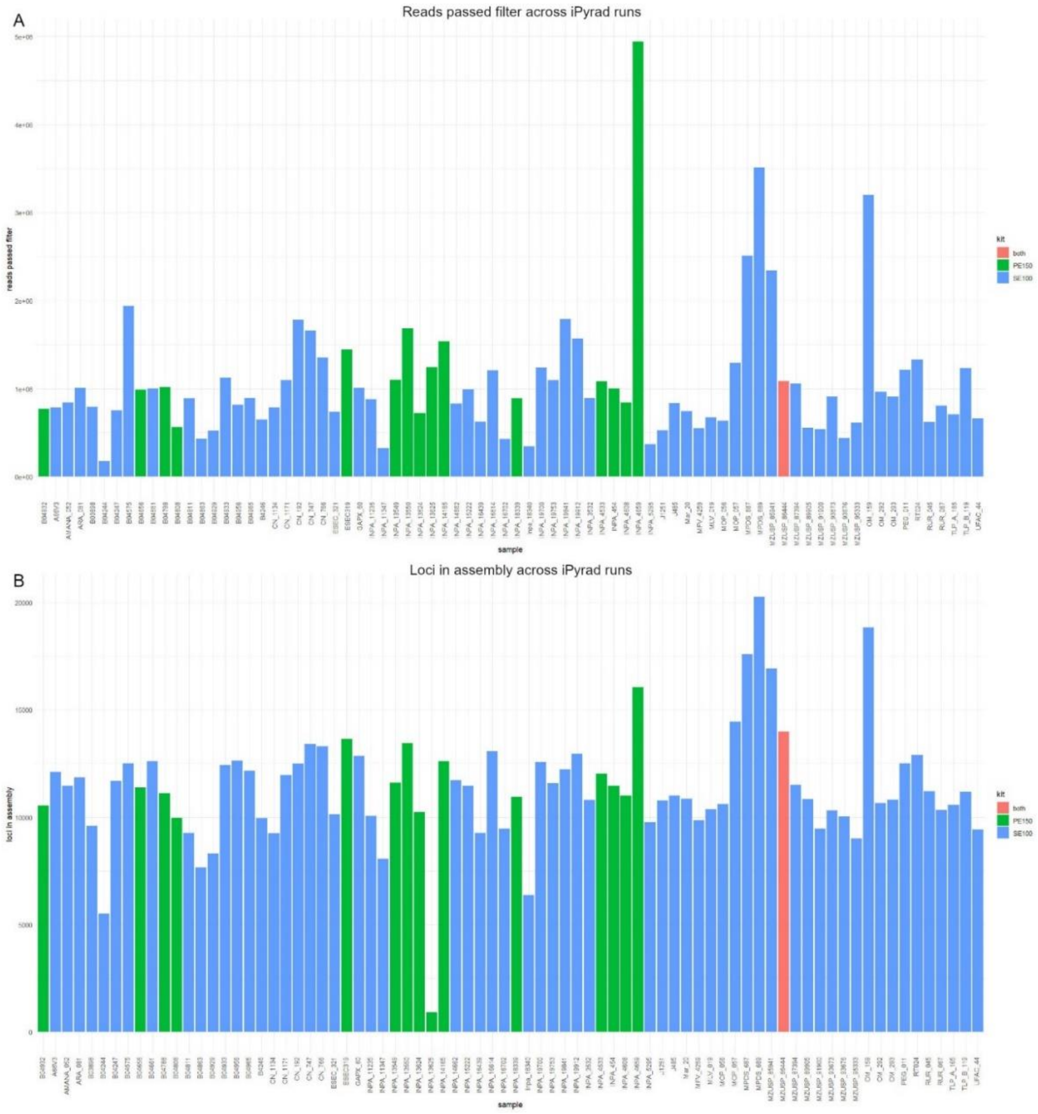

Figure S1 – Comparative results of ddRAD runs. The histograms are illustrating the results of different ddRAD runs, where kits PE150 (green) and SE100 (blue) were used, and a replica (red). (A) Number of reads passed filter across iPyrad runs. (B) Number of loci in assembly across iPyrad runs.
