# Supplementary material for: Phylogenomics of the gray-breasted sabrewing (Campylopterus largipennis) species complex in the Amazonia and Cerrado biomes
Source: Genet Mol Biol. 2024 Aug 5;47(3):e20230331. doi: 10.1590/1678-4685-GMB-2023-0331 (PMC11308382; doi:10.1590/1678-4685-GMB-2023-0331)
Supplement: Figure S3 - [file 1415-4757-GMB-47-3-e20230331-s3.pdf]

Genetics and Molecular Biology
https://doi.org/10.1590/1678-4685-GMB-2023-0331

Supplementary Material to "Phylogenomics of the gray-breasted sabrewing (*Campylopterus largipennis*) species complex in the Amazonia and Cerrado biomes"

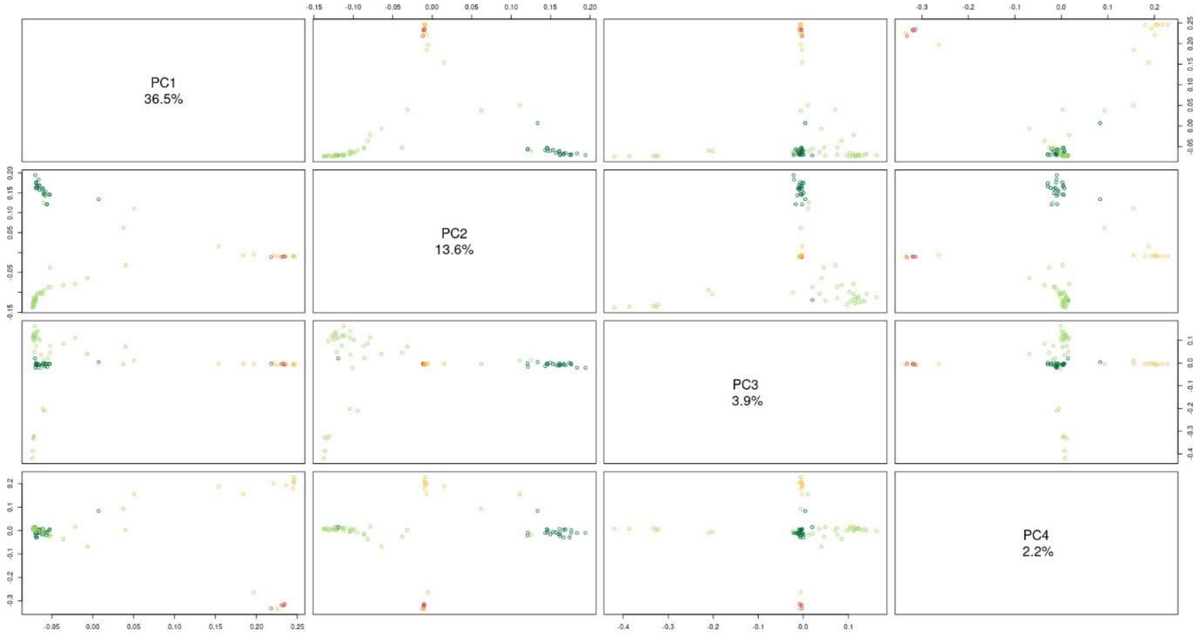

Figure S3 – Paired principal component analysis. Scatterplots showing the top four PCs. Population substructures can be observed among SEA samples in the PC3. A differentiation between CR and MS can be observed in the PC4. The groups are colored as follows: green for NWA, light green for SEA, yellow for CR, and red for MS.
