# Supplementary material for: Phylogenomics of the gray-breasted sabrewing (Campylopterus largipennis) species complex in the Amazonia and Cerrado biomes
Source: Genet Mol Biol. 2024 Aug 5;47(3):e20230331. doi: 10.1590/1678-4685-GMB-2023-0331 (PMC11308382; doi:10.1590/1678-4685-GMB-2023-0331)
Supplement: Table S1 - [file 1415-4757-GMB-47-3-e20230331-s5.pdf]

Genetics and Molecular Biology
https://doi.org/10.1590/1678-4685-GMB-2023-0331

Supplementary Material to "Phylogenomics of the gray-breasted sabrewing (*Campylopterus largipennis*) species complex in the Amazonia and Cerrado biomes"

Table S1 – Voucher specimens and location details of the samples belonging to the *Campylopterus largipennis* complex.

sample_id	Voucher	UF	locality	lat	long
A66V3	A66V3		Guyana (country)	6.766667	-58.166667
AMANÃ 052	MPEG 65112	PA	Itaituba	-5.352222	-57.475000
ARA 081	MPEG 72305	PA	Santarém	-2.783333	-55.600000
B03898	B03898	MG	Montes Claros	-16.735000	-43.861944
B04244	B04244	MG	Pico do Inficionado	-20.133333	-43.450278
B04246	B04246	MG	Santana do Riacho	-19.108431	-43.680697
B04247	B04247	MG	Serra do Cabral	-17.757778	-44.171944
B04575	B04575	MG	Olhos D'água	-17.558055	-43.736388
B04656	B04656	MG	Pico do Inficionado	-20.133333	-43.450278
B04661	B04661	MG	Pico do Inficionado	-20.133333	-43.450278
B04788	B04788	MG	Lassance	-17.844722	-44.418889
B04808	B04808	MG	Montes Claros	-16.646944	-43.934167
B04811	B04811	MG	Morro do Pilar	-19.257500	-43.525278
B04863	B04863	MG	Engenheiro Navarro	-17.388889	-43.895556
B04929	B04929	MG	Januária	-15.561389	-44.514444
B04932(472)	B04932	MG	Januária	-15.666389	-44.633056
B04933	B04933	RO	Porto Velho	-9.4544444	-64.371111
B04956	B04956	MG	Morro do Pilar	-19.257500	-43.525278
B04965	B04965	PA	Almerin	-1.522778	-52.581944
Cla_86444	MZUSP 86444	AM	Rio Parauari (Maués)	-3.856156	-57.747958
CN 1134	MPEG 66422	PA	Almerim	-1.522778	-52.581944
CN 1171	MPEG 66421	PA	Almerim	-1.522778	-52.581944
CN 192	MPEG 64604	PA	Faro	-1.700000	-57.200000
CN 747	MPEG 65737	PA	Oriximiná	1.283333	-58.683333
CN 766	MPEG 65738	PA	Oriximiná	1.283333	-58.683333
ESEC_319	MPEG 59773	AC	ESEC Rio Acre	-11.056668	-70.271112

sample_id	Voucher	UF	locality	lat	long
ESEC_321	MPEG 59774	AC	ESEC Rio Acre	-11.056667	-70.271111
GAPX 60	MPEG 73925	PA	São Félix do Xingu	-6.478611	-51.133889
INPA A 11235	INPA A 11235	PA	Tapajós, GM 2012 III	-4.853295	-56.699503
INPA A 11347	INPA A 11347	PA	Tapajós, GM 2012 III	-4.853295	-56.699503
INPA A 13549	INPA A 13549	AM	São Gabriel, 2013 MPEG	-0.150558	-66.800079
INPA A 13550	INPA A 13550	AM	São Gabriel, 2013 MPEG	-0.150558	-66.800079
INPA A 13624	INPA A 13624	AM	São Gabriel, 2013 MPEG	-0.150558	-66.800079
INPA A 13625	INPA A 13625	AM	São Gabriel, 2013 MPEG	-0.150558	-66.800079
INPA A 14165	INPA A 14165	AM	Anavilhanas, 2014	-2.278004	-60.840988
INPA A 14662	INPA A 14662	PA	Tapajós, Cassiano 2013	-5.233345	-56.885554
INPA A 15222	INPA A 15222	PA	Tapajós, Sisbiota 2013	-3.344145	-55.208515
INPA A 16439	INPA A 16439	PA	Trombetas, Sisbiota 2013	-1.430996	-56.792141
INPA A 16614	INPA A 16614	AM	PDBFF, North Manaus	-2.365803	-59.946276
INPA A 16702	INPA A 16702	PA	Trombetas, Sisbiota 2013	-1.430996	-56.792141
INPA A 18339	INPA A 18339	AM	Japurá, Sisbiota 2014	-1.723109	-69.139844
INPA A 18340	INPA A 18340	AM	Japurá, Sisbiota 2014	-1.723109	-69.139844
INPA A 19700	INPA A 19700	RR	Serra da Mocidade 2016	1.505789	-61.870967
INPA A 3532	INPA A 3532	RO	Rio Madeira 2010	-9.032130	-64.249477
INPA A 4533	INPA A 4533	AM	Jatapu, Sisbiota 2012	-1.916449	-58.299270
INPA A 454	INPA A 454	AM	Rio Madeira, Probio 2005	-6.296743	-60.359269
INPA A 4608	INPA A 4608	AM	Jatapu, Sisbiota 2012	-2.012698	-58.189202
INPA A 4659	INPA A 4659	AM	Jatapu, Sisbiota 2012	-2.012698	-58.189202
INPA A 5295	INPA A 5295	PA	Brasília Legal	-3.705664	-55.592373
J1251	MZUSP 108200	RO	Porto Velho	-9.441601	-64.851776
J485	MZUSP 108199	RO	Porto Velho	-9.628381	-65.057879
MAR 20	MPEG 57542	AM	Manicoré	-8.687222	-61.408056
MFV 4259	MFV 4259	MG	Jequitaí	-17.090001	-44.563888
MLV 019	MPEG 59057	PA	Santa Bárbara	-1.199167	-48.299167
MOP 056	MPEG 67120	PA	Ourilândia do Norte	-6.755000	-51.083889
MOP 057	MPEG 67119	PA	Ourilândia do Norte	-6.755000	-51.083889
MPDS 687	MPEG 58669	AM	Humaitá	-7.550000	-62.566667
MPDS 689	MPEG 58670	AM	Humaitá	-7.550000	-62.566667
MZUSP 85941	MZUSP 85941	PA	Curuá	-1.950036	-55.040847

sample_id	Voucher	UF	locality	lat	long
MZUSP 86444	MZUSP 86444	AM	Rio Parauari (Maués)	-3.856156	-57.747958
MZUSP 87394	MZUSP 87394	PA	Jacareacanga	-7.401039	-57.478419
MZUSP 89905	MZUSP 89905	PA	Santana do Araguaia	-9.556761	-50.602819
MZUSP 91900	MZUSP 91900	AP	Laranjal do Jari	1.012997	-53.182847
MZUSP 93673	MZUSP 93673	RR	Igarapé Caicubi	-0.589619	-62.167433
MZUSP 93676	MZUSP 93676	RR	Igarapé Caicubi	-0.589619	-62.167433
MZUSP 95333	MZUSP 95333	PA	Vitória do Xingu	-2.900236	-52.010481
OM 159	MPEG 71008	AM	Humaitá	-7.496667	-63.394722
OM 292	MPEG 71010	RO	Machadinho D'Oeste	-8.928333	-62.077500
OM 293	MPEG 71011	RO	Machadinho D'Oeste	-8.928333	-62.077500
PEG 011	MPEG 58639	PA	Santa Bárbara	-1.199167	-48.299167
RT024	MPEG 66083	PA	Parauapebas	-6.067778	-49.901944
RUR 045	MPEG 62281	AM	Coari	-4.850000	-65.066667
RUR 067	MPEG 62280	AM	Coari	-4.850000	-65.066667
TLP(A) 165	MPEG 67277	MT	Paranaíta	-9.665001	-56.476945
TLP(B) 119	MPEG 67276	MT	Paranaíta	-9.665002	-56.476946
UFAC 44	MPEG 59924	AC	Porto Acre	-9.756667	-67.670556
