# Supplementary material for: Phylogenomics of the gray-breasted sabrewing (Campylopterus largipennis) species complex in the Amazonia and Cerrado biomes
Source: Genet Mol Biol. 2024 Aug 5;47(3):e20230331. doi: 10.1590/1678-4685-GMB-2023-0331 (PMC11308382; doi:10.1590/1678-4685-GMB-2023-0331)
Supplement: Table S2 - [file 1415-4757-GMB-47-3-e20230331-s6.pdf]

Genetics and Molecular Biology
https://doi.org/10.1590/1678-4685-GMB-2023-0331

Supplementary Material to "Phylogenomics of the gray-breasted sabrewing (*Campylopterus largipennis*) species complex in the Amazonia and Cerrado biomes"

Table S2 – Pairwise FST values sampled localities The upper diagonal indicates the FST values and the confidence intervals are represented in the lower diagonal with lower and upper intervals, respectively.

	MS	CR	SEA	NWA
MS	-	0.2491598	0.5955114	0.6688105
CR	0.2204929 - 0.2768883	-	0.6255804	0.701648
SEA	0.5856418 - 0.6053551	0.6164746- 0.634206	-	0.2372886
NWA	0.6587404 - 0.6786075	0.6933207-0.7100213	0.2281272 - 0.2464998	-
