# Supplementary material for: Phylogenomics of the gray-breasted sabrewing (Campylopterus largipennis) species complex in the Amazonia and Cerrado biomes
Source: Genet Mol Biol. 2024 Aug 5;47(3):e20230331. doi: 10.1590/1678-4685-GMB-2023-0331 (PMC11308382; doi:10.1590/1678-4685-GMB-2023-0331)
Supplement: Table S3 - [file 1415-4757-GMB-47-3-e20230331-s7.pdf]

Genetics and Molecular Biology
https://doi.org/10.1590/1678-4685-GMB-2023-0331

Supplementary Material to "Phylogenomics of the gray-breasted sabrewing (*Campylopterus largipennis*) species complex in the Amazonia and Cerrado biomes"

Table S3 – Population parameters estimated from three independent runs of G-Phocs analysis considering the following relationship: ((NWA, SEA)(*CR*, *MS*)).

**Group/node**	**θ**	**τ**	**Ne=(θ/μ)/4**	**Divergence Time =τ/μ (HDP 95%)**
root	33.511	28.851	364250	1254.391304 (1131.769565 - 1334.582609)
Amazonia	33.911	14.851	368597.8261	645.6956522 (536.8086957 - 690.7347826)
Cerrado	4.268	4.672	46391.30435	203.1391304 (178.1869565 - 230.3434783)
NWA	81.44		885217.3913	0
SEA	119.099		1294554.348	0
CR	73.025		793750	0
MS	14.712		159913.0435	0
