# Supplementary material for: Phylogenomics of the gray-breasted sabrewing (Campylopterus largipennis) species complex in the Amazonia and Cerrado biomes
Source: Genet Mol Biol. 2024 Aug 5;47(3):e20230331. doi: 10.1590/1678-4685-GMB-2023-0331 (PMC11308382; doi:10.1590/1678-4685-GMB-2023-0331)
Supplement: Table S4 - [file 1415-4757-GMB-47-3-e20230331-s8.pdf]

Genetics and Molecular Biology
https://doi.org/10.1590/1678-4685-GMB-2023-0331

Supplementary Material to “Phylogenomics of the gray-breasted sabrewing (*Campylopterus largipennis*) species complex in the Amazonia and Cerrado biomes”

Table S4 – Migration bands from G-Phocs analysis.

Origin > Destination	Msx	θx	migrant proportion (msx × θx/4 = Msx)
Amazonia > Cerrado	28.882	4.268	27.06841612
Cerrado > Amazonia	29.987	33.911	3.537141341
NWA > SEA	27.999	119.099	0.9403605404
CR > SEA	29.445	119.099	0.9889251799
MS > SEA	28.98	119.099	0.9733079203
SEA > NWA	28.736	81.44	1.411394892
CR > NWA	29.668	81.44	1.457170923
MS > NWA	29.959	81.44	1.471463654
NWA > CR	30.3	73.025	1.65970558
SEA > CR	26.699	73.025	1.462458062
MS > CR	29.684	73.025	1.625963711
NWA > MS	29.248	14.712	7.952147906
SEA > MS	29.337	14.712	7.97634584
CR > MS	29.765	14.712	8.092713431
